# Customer Purchase Intentions and Choice in Food Retail Environments: A Scoping Review

**DOI:** 10.3390/ijerph15112493

**Published:** 2018-11-08

**Authors:** Iana A. Castro, Anuja Majmundar, Christine B. Williams, Barbara Baquero

**Affiliations:** 1 Marketing Department, Fowler College of Business, San Diego State University, San Diego, CA 92182, USA; 2 Department of Preventive Medicine, Keck School of Medicine, University of Southern California, Los Angeles, CA 90033, USA; anuja.majmundar@usc.edu; 3 Department of Pediatrics, University of California San Diego, San Diego, CA 92093, USA; cbwilliams2@gmail.com; 4 Department of Community and Behavioral Health, College of Public Health, University of Iowa, Iowa City, IA 52242, USA; barbara-baquero@uiowa.edu

**Keywords:** consumer health, obesity, community health, social marketing, health determinants

## Abstract

Food purchasing and consumption behaviors have implications for nutrition and obesity. Food retail environments, in particular, shape customer food choices and energy intake. The marketing literature offers insights about how public health practitioners can work within food retail environments to encourage healthy food choices. We reviewed experimental studies in the marketing literature to examine factors influencing customer purchase intentions and choice for food products in retail stores. Database searches were conducted in February 2016 for original, empirical articles published in English from 2000–2015 in marketing journals. Each research article included at least one experimental design study conducted in a real or simulated retail environment with purchase intentions or choice of food products as an outcome variable. Backward and forward reference searches were conducted for articles meeting inclusion criteria. Narrative synthesis methods were used to thematically group and summarize the findings of forty-one articles that met inclusion criteria into three categories: shelf display and product factors, pricing and price promotion factors, and in-store and customer decision-making factors. This research contributes to the literature by providing specific and actionable approaches that can increase/decrease customer purchase intentions and choice for food products in retail environments. Translating marketing strategies into public health applications can provide recommendations for future intervention research and policy related to customer food purchasing behavior.

## 1. Introduction

In the United States, an estimated 35% of adults and 17% of children are obese [1]. The influence of the food environment is significant for obesity rates. Recent work suggests that foods and beverages purchased in food retail stores contribute a significant portion (63%) of individual’s total daily energy intake and, among children, are the most important source of empty calories, added sugar, and solid fats [2,3,4]. Considering the impact of purchases made in food retail environments on diet intake and quality, and their implications for obesity and chronic disease, public health policymakers, practitioners, researchers, and community organizations are actively searching for effective strategies to promote healthier customer choices and purchases when shopping.

Food shopping behavior continues to evolve, and food retail environments are increasingly more complex, requiring a more in-depth understanding of customer behavior [5]. Food shopping occurs an average of 1.6 times per week and the responsibility for food shopping and preparation is shared among multiple members of a household [5]. Food retail environments, comprised of supermarkets, corner and convenience stores, supercenters, and ethnic and natural food stores, among others, may exert significant influence on food purchasing decisions and present opportunities for encouraging healthier food choices, given the majority of purchase decisions are made in the stores [6].

Existing evidence on healthy food retail strategies indicates an important gap in knowledge on how best to promote healthy food choices and purchases in food retail environments [7]. Progress is being made with evidence-based interventions focused on increasing access, promotion, and consumption of healthy foods [7], with most of the work thus far studying structural and social environmental changes. However, other in-store factors and customer-specific (i.e., intrapersonal) factors often examined in marketing research also influence customers’ in-store behavior. The aim of this scoping review is to provide a better understanding of customers’ in-store behavior, and factors that have an effect on purchase intentions and choice of food products, by reviewing articles published in the marketing literature. In effect, translating marketing strategies into public health applications may provide recommendations for interventions and policy related to customer food purchasing behavior [8].

## 2. Materials and Methods

The primary references in this scoping review were peer-reviewed articles published in marketing journals studying purchase intentions and/or choice in food retail environments. The American Marketing Association defines marketing as “the activity, set of institutions, and processes for creating, communicating, delivering and exchanging offerings that have value for customers, clients, partners and society at large” [9]. Understanding consumer behavior is an integral part of marketing, with researchers studying individuals, as well as groups and organizations, and the processes they use to select, secure, use, and dispose of products, services, and experiences to satisfy needs [10]. As such, the marketing literature can provide important insights into the factors that can influence customer purchase intentions and choice within food retail environments. Purchase intentions, in this study, are defined as intended behavior (e.g., likelihood to buy), while choice refers to the selection of a product. We maintain a specific focus on purchase intentions or choice to interpret learnings about purchase behaviors in a food context. Studies based on either purchase intentions or choice of food products only or both food and non-food products were included. We excluded studies examining these outcomes for non-food products only. The review focused on research using experimental design, either through field or laboratory studies (e.g., simulated store environments, scenario-based or simulated shopping tasks), for an in-situ customer understanding. Experimental design allows researchers to manipulate individual factors while controlling others in order to study and measure the effect of each factor on the outcome of interest [11]. This scoping review focused on studies using experimental design to capture differential effects of factors influencing purchase intentions and choice for future intervention research.

### 2.1. Search Strategy

The authors employed a three-step search strategy from February to July 2016, utilizing methods developed by Cooper (1998) [12]. For the first step, articles were identified for inclusion through comprehensive database literature searches on Web of Science Core Collection and ABI/INFORM. Web of Science Core Collection has the deepest citation coverage across the Sciences, Social Sciences, and Art and Humanities. ABI/INFORM indexes business publications. For each article included in the scoping review, a backward search of references was conducted, with each article listed in the references of the included article assessed for inclusion (step 2). Finally, a forward search was conducted for articles citing the included article (step 3) to identify any other relevant articles.

**Figure 1:**
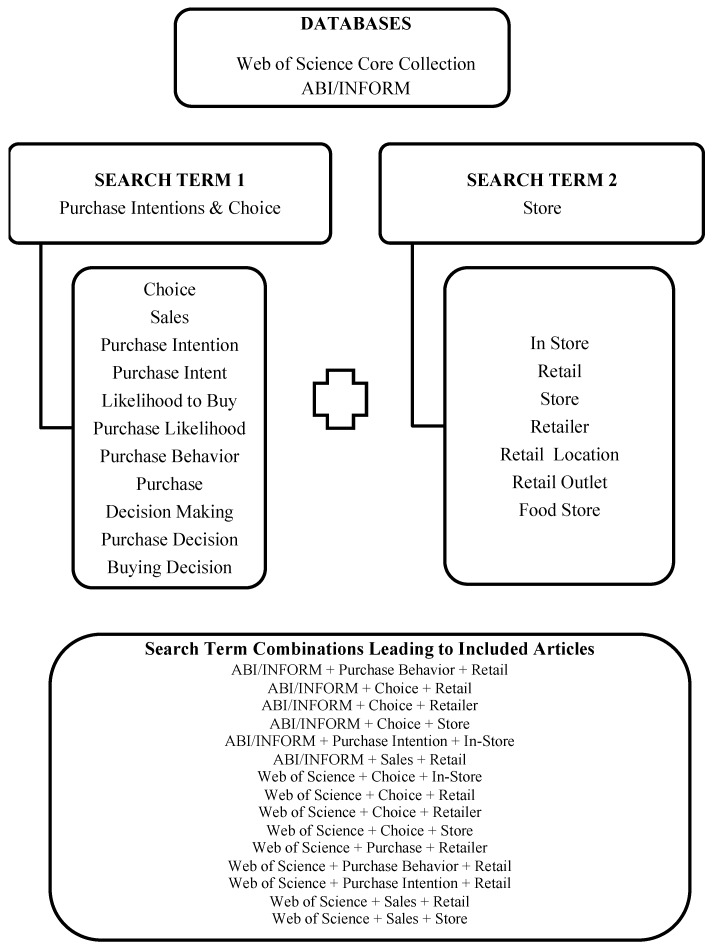
Databases, search terms, and search combinations leading to included articles.

For the database searches, an enhanced concept-to-operation correspondence was captured via multiple operationalism recommended for literature reviews [12]. We used a combination of search terms, one from each of two categories: (1) purchase intentions/choice and (2) store. A list of search terms for purchase intentions/choice and store was compiled (11 terms related to purchase intentions/choice, seven terms related to store). Each database search contained one term from each group for a total of 77 distinct searches per database (Figure 1). We queried each database with each unique combination of the search term pairs, using the operator ‘AND’ (e.g., retail + choice, in-store + choice, retail + purchase). Any search terms in article titles, abstracts or keywords were retrieved.

### 2.2. Inclusion Criteria

Search results were limited to articles (1) written in English, (2) published between 2000–2015, (3) original research conducted in any part of the world, and (4) published in peer-reviewed journals in marketing [13,14]. Results from each combination of search terms were downloaded, saved, and organized in unique reference libraries (Endnote version X7.5.1, Thomson Scientific, Philadelphia, PA, USA). Articles underwent three rounds of screenings to assess inclusion criteria conducted by two study authors. For each round in the screening process, decisions were based on the inclusion and exclusion criteria detailed in Table 1. Three rounds of screening were necessary to efficiently and accurately determine what articles met inclusion criteria given the number of articles emerging from the searches and the number of studies within each article.

### 2.3. Data Extraction and Synthesis

Articles published in marketing journals frequently contain multiple studies in one article. Data extracted from relevant studies that met inclusion criteria included manipulated variables and conditions, type of experiment, outcome variables, and key findings. In order to summarize and disseminate the research findings, narrative synthesis was used to thematically group and report findings in the manuscript and a table was created that captured detailed information from each article and serves as a reference for future research (Table 2).

## 3. Results

**Figure 2:**
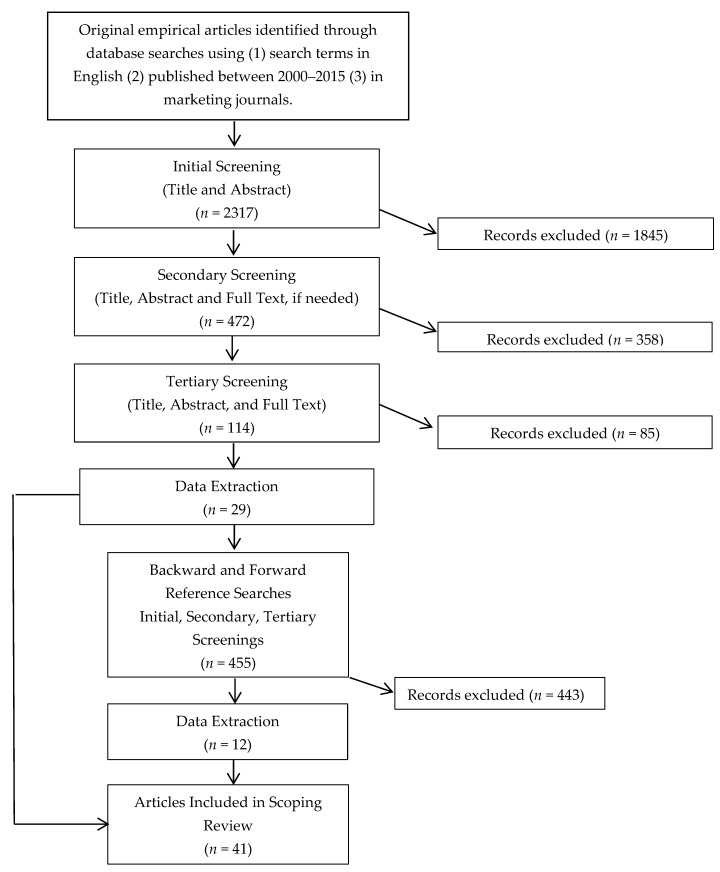
Article Inclusion Flow Chart.

This scoping review focused on studies that employed experimental research designs, in which individual factors are manipulated in order to study and measure the effect of each manipulation on the outcome of interest [11]. Twenty-nine articles met the final detailed inclusion criteria of: at least one study in the article (1) was conducted in a real or simulated retail setting, (2) used experimental design, (3) included purchase intentions or choice as the outcome variable and (4) had food as the product(s) tested. Backward and forward searches of included articles resulted in an additional 12 qualified articles for a total of 41 articles (Figure 2).

Each article included an average of three studies (range: 1–5); the majority of articles had one or two studies that met inclusion criteria. Please refer to Table 2 for a summary of included articles, including the manipulated variables and conditions, type of experiment, outcome variables, and a brief summary of key findings. Type of experiment was categorized as either laboratory or field. Laboratory experiment procedures varied and included asking participants to complete a shopping task, to imagine a scenario and behave as they would in that situation, or to consider products in a simulated store or arranged on a shelf, among others. Field experiments were conducted in real-world retail environments, including grocery stores and small food stores. Outcome variables included purchase intentions, hypothetical choice (i.e., participants indicated what they would select), and choice (i.e., participants made a selection and received the product). The narrative synthesis of results presented below thematically groups and summarizes findings based on three types of factors: shelf display and product factors, pricing and price promotion factors, and in-store and customer decision-making factors. The focus of the synthesis is on discussing factors that were shown to have an effect on purchase intentions or choice.

### 3.1. Shelf Display and Product Factors

#### 3.1.1. Shelf Display

Customer choice and purchase intentions can be influenced by product location on the shelf, the appearance of the products on the shelf, the brands available, and product attributes. Shelf display and product factors can capture customers’ attention and serve as sources of information for customers when making purchase decisions [15,16,17,18,19]. At the shelf display level, location can impact the visual attention a product receives, and attention is correlated with choice [16,17,18]. Products located in the horizontal center of the product category receive more attention and are more likely to be chosen [17]. At the product level, product packaging is associated with purchase intentions [19], and when a customer repeatedly allocates attention to a product, he/she is more likely to select the product in a subsequent choice [18].

Customer choices are also associated with the inferences customers draw when looking at shelf displays. Product display factors, such as shelf-based scarcity (i.e., few products left on the shelf) can create positive inferences regarding the popularity and quality of the products on display, leading to higher purchase intentions for those products [20,21,22]. In contrast, shelf display conditions can negatively affect purchase intentions and choice for familiar brand food products if display quantity and disorganization suggest others have touched the products [21].

#### 3.1.2. Branding

Brand familiarity and availability can also impact purchase intentions and choice. Brand familiarity is important as customers are more likely to choose familiar over unfamiliar brands [21]. Customers’ willingness to buy new private label brands is dependent on the product categories and can be shaped by the financial, functional and social risks associated with the product category [23]. In the case of private label brand products, mimicking the packaging of the national brand (versus not) can lead to inferences that the private label products are of higher quality and of similar origin as the national brand, resulting in higher purchase intentions [24]. At the product level, priming customers with a specific brand that is related to a current need can increase choice for the brand (e.g., priming a drink brand when the customer is thirsty) [25]. Brands with multiple products in a product line benefit from a strong brand reputation [26], and brands that successfully focus on specific social causes find an optimal balance between the brand, cause, and customer social identity [27]. Finally, product attributes such as product shape, size, and color can impact customer purchase intentions and choice. Even attributes that are considered to be unnecessary, such as the color of a soft drink, can steer choices when customers are faced with a set of options and cannot decide on one based on important attributes alone [28].

#### 3.1.3. Nutrition Labeling

Providing health information on product packaging can positively or negatively influence purchase intentions and choice based on customer knowledge [29,30]. High customer motivation to process nutrition information and high customer knowledge led to lower purchase intentions for less healthy products (i.e., high trans-fat). However, purchase intentions for less healthy products are highest for customers high in customer motivation to process nutrition information and low in customer knowledge, suggesting that without knowledge, customers are unable to properly interpret the information [29]. However, providing objective information about the products can help inform customers. For products for which customers have pre-conceived biased impressions (e.g., an unhealthy product is perceived as healthy or a healthy product is perceived as unhealthy), providing objective information can increase or decrease choice of the products. In other words, providing objective information that a product that is perceived as healthy is actually unhealthy, can reduce choice of the product, while providing information that a product perceived to be unhealthy is actually healthy can increase choice of the product. When the information confirms the bias, differences in choice do not emerge [31].

Front-of-package labeling systems can also provide customers with information that can influence decision-making. Customers use front-of-package labeling systems, including reductive labels (i.e., providing calories and three nutrients to limit) and evaluative labels (e.g., an icon indicating the product is healthy or the product’s level of healthfulness) differently. Evaluative labels are more beneficial when customers are comparing products, while reductive labels are more effective when evaluating a single product [32]. Finally, front-of-package labeling can play a role in children’s choices. A study focused on children found that that the presence of an on-package claim (e.g., “Cereal that’s good for you”) in the choice set led to unhealthy choices. This shift appeared to occur particularly when the claim appeared on healthier products [33].

#### 3.1.4. Food Sampling

Food samples can impact the healthfulness of products purchased [34,35]. Offering customers a healthy food sample, or one that is framed as healthy, can lead to more purchases of fruits and vegetables and healthier choices during the shopping trip. Conversely, customers who were offered unhealthy food samples made more unhealthy choices [35]. Distractions while sampling, such as having a shopping list memorized, also lead to higher choice of the sampled products [34].

### 3.2. Pricing and Price Promotion Factors

Price and promotion information, and how it is presented, can influence purchase intentions and choice. Unit price information can lead customers to choose products with lower unit prices [36], since it makes prices more salient, increasing customers’ price sensitivity [37]. However, multiple unit price promotions (e.g., 8 for $8) increase purchase quantities for higher (e.g., Gatorade) vs. lower (e.g., ketchup) consumption products, with higher numbers leading to more purchases than lower numbers (e.g., 8 for $8 vs. 2 for $2) [38]. In terms of wording for price promotions, repeating initial word sounds across two or more words that are close together can steer product choice by facilitating message processing (e.g., 2 Twix $2) [39]. In terms of framing for price promotions, providing customers with comparative price information (e.g., sale price and regular price) can influence purchase intentions and choice. Comparative price information should be presented in absolute values when discount size is small and in percentage terms when discount size is large to increase purchase intentions [40]. However, customers do face computational errors when processing percentages [41]. Finally, customers who used a surprise in-store coupon for a planned purchase made more unplanned purchases of treat items, items that were cognitively related to the one primed by the coupon, and items shelved near the one primed by the surprise coupon. They also spent more than those who did not get the coupon and more than the discount from the coupon [42].

Price promotions can negatively influence sales of products after the sale period has ended. Promotions that offer large percentage off (versus cents off) discounts negatively influence customers’ purchase intentions for the brand after the sale [43]. Additionally, when customers are aware of the fact that they missed a sale, they are more likely to buy the product for a less attractive discount than use unexpected and free money (such as a cash gift from a friend) to pay full price, since they feel it is not a good deal to pay full price regardless of how much unexpected and free money was received [44].

Customers’ responses to price promotions are based on individual differences, including their decision-making styles (i.e., analytical or intuitive) and regulatory focus (i.e., prevention or promotion). Analytical decision-makers focus on both utilitarian and hedonic benefits, and intuitive decision-makers focus on hedonic benefits, influencing likelihood to buy [45]. Additionally, promotion framing that aligns with customers’ regulatory focus may lead customers to purchase more products that are not being promoted, affecting the overall basket [46]. Ultimately, the ability of price promotions to get a customer to switch from one brand to another depends on the customer’s consideration set, the price-quality tiers of the brands, the make-up of the choice set, and individual differences [47].

### 3.3. In-Store and Customer Decision-Making Factors

Both intrapersonal factors and in-store environment factors can impact the customer’s experience while in the store and shape customer purchase intentions and choice [48,49,50,51,52,53,54,55]. Customers’ implicit beliefs about the relationship between taste and healthfulness, bringing reusable bags to the store, making multiple choices in a row, receiving real-time feedback on spending while on a budget, and paying with a credit/debit card are all linked to less healthy choices [48,49,50,51,52]. One of the articles found that unhealthy foods are perceived to be tastier, are rated as more enjoyable during consumption, and were preferred in a choice task when an enjoyment goal (vs. no goal) was activated; therefore, customers will buy less healthy products when focused on enjoyment [48]. Bringing reusable bags to the store increased purchase intentions and choice for unhealthy products due to licensing, with customers rewarding themselves for doing something good (e.g., I did something good for the environment by bringing reusable bags, so I deserve a treat) [49]. Being forced to bring your own reusable bags (e.g., store requirement), having dependents at home, or focusing on the price of the products attenuates this result [49].

The processes that customers engage in while shopping may also increase unhealthy choices. When customers make multiple choices in a row when grocery shopping (vs. following purchase instructions, such as a shopping list with specific product and brand information), their self-control weakens, leading to unhealthy subsequent choices [50]. For customers who are on a budget, getting real-time spending feedback (i.e., customers see the price of their overall basket every time an item is added) reduces the mental stress of keeping track of spending and leads to less healthy product purchases while increasing spending without going over budget. However, shoppers without a budget spend less as a result of real-time spending feedback [51]. Finally, paying with a credit or debit card (vs. cash) leads to more unhealthy product purchases because paying with cash is more ‘painful’ [52].

Store environment factors also steer behaviors. When customers feel confined (e.g., smaller aisles) they make more varied choices [54], and a simple (vs. complex or no) scent in the environment increases the number of items selected from product categories that do not contain required purchases rather than from required categories [55].

## 4. Discussion

The findings of this review build on existing intervention research in the field of public health [7,56,57,58,59,60,61] by drawing from the marketing literature and focusing exclusively on studies that employed experimental design, were conducted in retail environments, and focused on customer purchase intentions and choice for food products as outcome variables. The review offers specific and actionable approaches that can be used to increase/decrease customer purchase intentions and choice for food products in retail environments. Prior research in public health has implemented interventions focused on increasing access, promotion, and consumption of healthy foods [7,56,57]. Our results contribute to existing research by providing specific marketing techniques related to shelf display and product factors, pricing and price promotions factors, and in-store and customer-specific factors that have been shown to influence purchase intentions and choice (see Table 2) and can be implemented in future intervention research focused on improving food decision-making in the environments where decision-making is taking place.

Some intervention strategies can begin before the customer enters the store and others can be implemented to facilitate healthier choices while shopping. Intervention strategies can focus on educating customers on how to prepare for the shopping experience. The United States Department of Agriculture (USDA) Supplemental Nutrition Assistance Program Education (SNAP-Ed) teaches program participants about nutrition and how to stretch their food dollars. Existing recommendations include planning ahead to know what is needed, making a shopping list, and knowing how much is available to spend [62]. Our findings suggest that in addition to asking customers to make a shopping list, customers can be encouraged to create a shopping list that includes specific details about products to be purchased to reduce the number of in-store choices [50]. Further, in addition to having a budget, customers can be asked to set a savings goal for the shopping trip and bring cash to pay for the purchase, all of which can reduce unhealthy purchases [49,51,52]. A health implication of a cash payment strategy is to encourage ‘audit’ behavior rooted in monitoring an upper limit on spending that invariably involves constant re-evaluation of the shopping basket to exclude indulgent/impulsive purchases, which often tend to be less healthy.

Store-based interventions have focused on point of purchase information, price promotion strategies, and healthy food accessibility and availability [7,56,57,58,59,60,61]. Point-of-purchase activities and materials (e.g., food demonstrations; taste tests; printed materials, such as recipe cards) are commonly used strategies in supermarkets and in supermarket interventions [7]. Retailers use food demonstrations and taste tests to introduce customers to new products, encourage sampling, and increase sales of sampled products. A number of findings from this review can contribute to future efforts using point-of-purchase strategies to increase healthy choices. For example, providing healthy food samples to customers as they enter the store and, importantly, allowing customers to enjoy the sampling experience while being distracted [34,35], can increase healthy choices and improve the healthfulness of the overall shopping basket [35]. The timing of offering the healthy sample and incorporation of distractions are new learnings from an intervention standpoint. These insights have direct relevance for interventions that promote healthy eating by means of simple food preparation and sampling in-store. Additionally, sampling may also help decrease the number of choices made based on the unhealthy-equals-tasty belief by providing samples of healthy products that are tasty [48]. One way of situating these learnings in retail environments is by providing healthy samples in the produce section, which in most cases is located near the front of the store and providing recipes that incorporate sampled products in the prepared foods section of the store.

Past intervention research has implemented pricing strategies (e.g., incentives, rebates, discounts and coupons) [7,60,61]; however, our findings suggest that the framing, timing and format of such strategies must be considered to understand the impact of the price promotion on customers’ buying decisions [36,37,38,39,40]. For example, surprise in-store coupons for planned purchases result in overspending and unplanned purchases [42]. Additionally, surprise coupons also increase purchases of products that are cognitively related to or located in close proximity to the discounted product. Therefore, a surprise coupon for a healthier item could lead to healthier purchases. When considering discounts, how a discount is presented can influence purchase intentions and choice and should be based on the size of the discount; however, it is important to consider that customers tend to struggle with calculating discounts based on percentages (e.g., translating a 15% off sale to actual dollar discount for an item). Past research establishes that sensitivity for healthy products is higher for price increases than decreases, and the opposite holds true for unhealthy foods [63]. Pricing strategies discussed in this review can be instrumental in decreasing demand sensitivity and thereby promoting unhealthy to healthy food choice switches among customers.

Research has addressed ways of nudging consumers toward healthier choices through product placement, increasing the salience of food accessibility and availability [58]. The findings suggest that product and shelf display strategies in the store can focus on attracting attention, given that increased attention to a product is correlated with choice. Attention can be increased by means of horizontal center product placement and product placement can be used to increase visibility for healthier options within the product category [17]. This has important implications for interventions aimed at boosting demand for local brands, unfamiliar brands, new products, and healthy products or produce (non-branded). Such strategies could benefit both retailers and customers by familiarizing customers with products, given that familiar brands and products are more likely to be selected than unfamiliar ones. Product placement can also be used to expose customers to products they did not realize the store carried. This can be an important strategy for small- and medium-sized food stores that are carrying new products due to the USDA’s new stocking requirements for stores that accept SNAP [64]. New products should be prominently displayed and strategically placed so that customers are made aware of the accessibility and availability of new products. The findings of the review can also serve to inform technical assistance programs that are funded by federal and private grants and are being created and implemented to promote healthy food access in underserved communities by working with food retail stores [65].

Insights on the effectiveness of nutrition labeling on packaging also raise important policy implications. In May 2016, after 20 years of the same nutrition label, the Food and Drug Administration (FDA) released a new nutrition label design [66]. The new FDA policy will require testing in research and practice to understand if and how the new labels affect customer choice and purchase intentions. Moreover, these changes need to be tested among diverse populations with different education levels, socio-economic status, cultural norms, and geographic locations. Situating findings from this review in the emerging policy context, there is a need for educating customers, especially those not as motivated or those that are motivated but lack knowledge, to search for and interpret nutritional information provided in the newly introduced nutrition labels [29,30]. Additionally, front-of-package labels can help inform consumers [59]. The findings of this review suggest that labels that provide both specific information about how the product performs on key nutritional values (e.g., calories and fat) and those that simply let customers know that a product is deemed ‘healthy’ can enable decision-making [32]. Additional research in this area is pertinent to respond to FDA’s recent request for information on ways to redefine the term ‘healthy’ as a nutrient content claim that is consistent with dietary recommendations, facilitates quick and healthy food choices, and regulates food products marketed as ‘healthy’ [67]. Given that biased impressions of products often lead to suboptimal food choices, providing objective, easily understandable information can improve healthy decision-making [31].

A key strength of the current review is the inter-disciplinary approach to understanding food purchases and health. By reviewing articles in the marketing discipline, our goal is to motivate evidence-based marketing practices to be translated into public health applications. This project was also a collaboration between public health and marketing researchers. Public health researchers provided critical inputs during the research conceptualization and manuscript development process to contextualize marketing insights in a public health framework. Marketing researchers, in turn, lent their domain expertise during data extraction and synthesis and during manuscript preparation. The current review maximizes the opportunity for the inter-disciplinary exchange of ideas and knowledge to achieve better health outcomes for communities.

The search for articles was limited to peer-reviewed journals in English, which may introduce a publication bias if published articles do not represent marketing scholarship investigating choice and purchase intentions. Additionally, the search for articles was conducted in 2016, so the review does not include articles that may meet inclusion criteria published after the search dates. In order to synthesize findings to understand consumer behavior, we limited the review to include laboratory and field experiments with purchase intentions or choice of food products as the outcome variables. In doing so, a number of articles with relevant outcome variables and settings were excluded because they were based on secondary panel data, sales/scanner data, survey/interview data, qualitative data, and/or did not include food products. While findings from these studies may further expand understanding of customer choice and purchase intentions, the emphasis on laboratory and field experiments as a first step allowed us to focus on understanding how controlled manipulations directly affect these outcome variables. Additionally, we are unable to relate the findings to socio-economic disparities. Future public health interventions can assess the implications of these findings given different socio-economic contexts. Despite these limitations, the strengths of this review include drawing a bridge between marketing insights and actionable strategies for healthy food-related interventions and identifying future avenues of research for obesity and nutrition interventions.

## 5. Conclusions

While findings reveal how different factors affect customer buying behavior, future studies must explore how these translate into long-term and sustainable consumer behavior change, including changes to actual consumption and repeat purchases over time. Understanding how sampling, nutrition labeling, shelf and product displays, and pricing and promotions impact behavior in the food retail environment may help policy makers understand and prioritize strategies to promote customer choice of healthy items. A series of longitudinal, randomized controlled trials to examine the interaction of these variables in food retail environments over time would allow researchers to tease out the most important factors, and make evidence-based policy recommendations based on the most influential and cost-effective factors. Future studies must examine varied theoretical approaches as well as culture, geographic location, socioeconomic status, and other demographics and how they may influence customer choice in a variety of large and small food retail settings. Finally, the marketing literature provides theories that may be useful in further understanding customer behavior in food retail environments. Future research can review and incorporate consumer behavior theories into intervention development.

## Figures and Tables

**Table 1:** Inclusion and exclusion criteria for each round of screening.

Initial Screening (Title and Abstract)		
	Inclusion Criteria	Exclusion Criteria
Type of Study	- Quantitative	
Environment	- Conducted in real or simulated ecommerce or physical retail environment, including scenario-based, retail as context or using secondary data from retailers	
Outcome Variables	- Choice or purchase intent	
Secondary Screening (Title, Abstract and Full Text, if needed)		
	Inclusion Criteria	Exclusion Criteria
Type of Study	- Experimental - Surveys or interviews - Secondary data	- Focused on providing insight into retailer operations or business-to -business practices, not business-to-consumer
Environment		- Online or ecommerce only - Interplay between online or ecommerce and brick and mortar
Outcome Variables		- Choice or purchase intent of channel or store choice - Nonfood products only
Tertiary Screening (Title, Abstract, and Full Text)		
	Inclusion Criteria (if at least one study met criteria)	Exclusion Criteria
Type of Study	- Experimental design	- Survey or interviews only - Secondary data only - Focuses on developing model of behavior
Environment	- Conducted in real or simulated physical retail environment	
Outcome Variables	- Choice and/or purchase intentions as key outcome variable	

**Table 2:** Summary of studies.

**Shelf Display and Product Factors** **(e.g., Product Location on the Shelf, Shelf Display Organization, Quantity of Product on the Shelf, Brand Familiarity, Product Packaging, Nutrition Labeling)**				
**Article Title (Reference)**	**Manipulated Variables (Conditions)**	**Experiment**	**Outcome Variables**	**Key Findings**
The influence of in-store product holders on orientation towards the product and on purchase intention. [15]	- Brand awareness (low, medium, high) - Product holder (none, shelf, display stand)	- Laboratory	- Purchase intentions	Brand awareness influenced purchase intentions. Product holder did not directly influence purchase intentions.
Decisive visual saliency and consumers’ in-store decisions. [16]	- Product location (private label and national brand on the same level, private label and national brand on different levels) - Product signage (with, without)	- Laboratory - Field	- Hypothetical choice - Choice	Visual attention is associated with choice. Optimal placement and signage can influence customer attention and choice.
Shining in the center: Central gaze cascade effect on product choice. [17]	- Product location within category (left, center, right) - Product category location on shelf (left, right)	- Laboratory	- Hypothetical choice	Horizontal centrality is related to visual attention and choice.
The influence of selective attention and inattention to products on subsequent choice. [18]	- Attention (selectively attended, unattended) - Instruction generalization (select, remove) - Choice task (designated vs. neutral, neglected vs. neutral) - Search screen display format (separated, side-by-side) - Search screen (two-item with a neglected vs. neutral alternative choice task, two-item with a designated vs. neutral alternative choice task, one-item with a designated vs. neutral alternative choice task)	- Laboratory	- Choice - Hypothetical choice	Selective attention and inattention influence choice between focal products and competing products.
Cutting through the clutter: purchase intentions as a function of packaging instrumentality, aesthetics, and symbolism. [19]	- Packaging (opaque, transparent)	- Field	- Purchase intentions	Opaque packaging was associated with higher purchase intentions than transparent packaging.
When shelf-based scarcity impacts consumer preferences. [20]	- Relative scarcity (option A more scarce than B, option B more scarce than A) - Purchase (for self, for others) - Scarcity (scarce, abundant) - Sales ranking (higher, lower) - Scarce option quality (high, low) - Abundant option quality (high, low)	- Laboratory	- Hypothetical choice - Choice	Scarce products are generally more preferred and shelf-based scarcity can influence choice of products based on customer inferences and the information available.
The influence of disorganized shelf displays and limited product quantity on consumer purchase. [21]	- Shelf display organization (organized, disorganized) - Product quantity (one, many) - Brand familiarity (familiar, unfamiliar) - Shelf display (one item disorganized, fully stocked and organized) - Shelf display (one item disorganized, a few items disorganized, fully stocked and organized)	- Laboratory - Field	- Purchase intentions - Choice	For familiar brand food products, disorganized displays with limited product quantity reduce purchase likelihood and choice.
Scarcity polarizes preferences: the impact on choice among multiple items in a product class. [22]	- Product scarcity (scarce, abundant) - Background (arousing, control)	- Laboratory	- Hypothetical choice	Product scarcity salience is positively associated with product choice.
Customers’ willingness to purchase new store brands. [23]	- Price advantage against leading national brand (10%, 20%, 40%) - Product quality positioning (classic, generic, premium store band)	- Laboratory	- Purchase intentions	Customers’ willingness to buy new store brands is lowest for product groups associated with high social risk and premium store brands are preferred for these categories.
The impact of copycat packaging strategies on the adoption of private labels. [24]	- Private label packaging strategy (copycat, original) - Product category (hedonic, utilitarian)	- Laboratory	- Hypothetical choice	Customers are more likely to choose private label products when they follow a copycat (vs. original) packaging strategy.
Drink Coca-Cola, eat popcorn, and choose Powerade: testing the limits of subliminal persuasion. [25]	- Brand priming (focal, neutral) - Thirst (low, high)	- Laboratory	- Choice	Priming effects can affect choice but are limited in duration.
The interplay of products from the same product line: the role of brand reputation. [26]	- Value brand (store brand, national brand)	- Laboratory	- Hypothetical choice	Presence of a value store brand caused an increase in the choice share of the premium store brand.
Attention, emotions and cause-related marketing effectiveness. [27]	- Product type (hedonic, utilitarian) - Cause (related, unrelated) - Cause fit (high, low)	- Laboratory	- Hypothetical choice	Emotional arousal, pleasure and attention to the cause are important when understanding altruistic choices.
Why is the trivial important? A reasons-based account for the effects of trivial attributes on choice. [28]	- Set size (2 brands, 3 brands) - Trivial attribute (present, absent) - Trivial attribute condition (no brands with a trivial attribute, target brand with trivial attribute, two alternative brands with trivial attributes, all brands with trivial attributes)	- Laboratory	- Hypothetical choice	Trivial attributes become significant in a purchase process when consumers face a decision problem.
How modification of the Nutrition Facts Panel influences consumers at risk for heart disease: The case of trans fat. [29]	- Nutrient content claim (low trans-fat, low trans-fat with health claim, no claim) - Knowledge (induced, not induced)	- Laboratory	- Purchase intentions	Customers high in motivation but lacking in knowledge had the highest purchase intentions of all groups for products high in trans-fat, highlighting the importance of knowledge in interpreting nutrition information.
Hold the Salt! Effects of sodium information provision, sodium content, and hypertension on perceived cardiovascular disease risk and purchase intentions. [30]	- Sodium content level (high, low) - Health related sodium educational materials (provided, not provided)	- Laboratory	- Purchase intentions	Providing customers with information about sodium levels decreases purchase intentions for high sodium products.
Broken halos and shattered horns: overcoming the biasing effects of prior expectations through objective information disclosure. [31]	- Nutrition facts panel (absent, present) - Product type (less healthful confirming health horn, more healthful inconsistent with health horn, more healthful consistent with health halo, less healthful inconsistent with health halo) - Nutrition disclosure condition (nutrition information absent, nutrition information provided on package, nutrition information on a poster)	- Laboratory	- Hypothetical choice - Purchase intentions	Objective nutrition information can influence purchase intentions and choice, especially when health biases are disconfirmed by the information provided.
Shopper response to front-of-package nutrition labeling programs: Potential consumer and retail store benefits. [32]	- Front of package label (reductive label system, evaluative label system using a single icon, both reductive and evaluative label systems, control condition with no labeling system) - Evaluative icon (Healthy Stars, control) - Reductive icon (Facts Up Front, control) - Objective product healthfulness (more healthful, less healthful)	- Laboratory	- Purchase intentions - Hypothetical choice	Front of package labeling systems can influence purchase intentions and choice. Evaluative systems are more beneficial when comparing products, while reductive systems are effective when evaluating a single product. Offering both may be the best way to assist customers when making decisions.
Children’s use of on-package nutritional claim information. [33]	- On package claim type (none, nutrient content claim, health claim, general claim) - Curriculum (before, after)	- Laboratory	- Hypothetical choice	Claims on packages can have a negative effect on children’s choices.
The influence of consumer distractions on the effectiveness of food-sampling programs. [34]	- Distraction (high, low) - Valence of informational component (low, high) - Preload (salt, sweet)	- Laboratory	- Choice	Distractions can lead to higher choice of the sampled product.
An apple a day brings more apples your way: Healthy samples prime healthier choices. [35]	- Sample (apple, cookie, none) - Sample (healthy/low calorie, unhealthy/high calorie) - Framing of sample (healthy, indulgent, no sample control)	- Laboratory - Field	- Choice - Hypothetical choice	Offering food samples that are healthy or perceived to be healthy leads to healthier choices.
**Pricing and Price Promotion Factors** **(e.g., Unit Price Information, Price Promotion, Promotion Framing)**				
**Article Title (Reference)**	**Manipulated Variables (Conditions)**	**Experiment**	**Outcome Variables**	**Key Findings**
Unit prices on retail shelf labels: An assessment of information prominence. [36]	- Unit price information prominence (low, high)	- Laboratory	- Hypothetical choice	More prominent unit price information shifts consumer choice to products with lower unit prices.
Unit pricing increases price sensitivity even when products are of identical size. [37]	- Unit price (present, absent)	- Laboratory	- Hypothetical choice - Purchase intentions	Displaying unit prices increases the salience of price and consumers are motivated to select lower-priced products.
Multiple unit price promotions and their effects on quantity purchase intentions. [38]	- Number of units in price promotion (2, 4, 8) - Single unit price information (present, absent) - Price promotion ($1 each, 8 for $8)	- Laboratory	- Purchase intentions - Hypothetical choice	Multiple unit price promotions increase quantities purchased.
Alliteration alters: Phonetic overlap in promotional messages influences evaluations and choice. [39]	- Pricing presentation (alliterative, non-alliterative, partially non-alliterative)	- Laboratory	- Hypothetical choice	Alliterative price promotions can influence product choice, even when the non-alliterative pricing is objectively better.
Effects of discount framing in comparative price advertising. [40]	- Discount size (small, large) - Discount framing (percentage, absolute)	- Laboratory	- Purchase intentions	Discount size and discount framing have an effect on purchase intentions in the case of comparative price promotions.
When more is less: the impact of base value neglect on consumer preferences for bonus packs over price discounts. [41]	- Type of promotion (bonus pack, price discount)	- Field	- Choice	More purchases occur for bonus packs over economically equivalent price discounts.
Pleasant surprises: Consumer response to unexpected in-store coupons. [42]	- Coupon (unexpected in-store coupon for item customer was planning on buying, no coupon)	- Field	- Choice	Giving customers a surprise coupon for a planned purchase led customers to buy more products. Customers spent more than the value of the coupon.
Cents or percent? The effects of promotion framing on price expectations and choice. [43]	- Promotion frame (percentage off, cents off) - Promotion depth (high, low)	- Laboratory	- Hypothetical Choice	Promotion frame and depth can influence choice and price expectations for products.
How consumers value transactions that entail using windfall money to offset missed price discounts. [44]	- Discount or windfall money ($25 discount, $25 windfall cash, $50 windfall cash, $75 windfall cash) - Unexpected money (store gift card, windfall cash)	- Laboratory	- Purchase intentions	When customers miss a prior discount, they are more likely to buy the product at a discount than pay full price with windfall money.
Utilitarian and hedonic promotional appeals of 99-ending prices: The influence of decision-making style. [45]	- Decision-making style (intuitive, analytical)	- Laboratory	- Purchase intentions	Decision-making style (i.e., analytical vs. intuitive) will influence customers’ likelihood of buying products priced with 99 endings.
The effect of sales promotions on the size and composition of the shopping basket: Regulatory compatibility from framing and temporal restrictions. [46]	- Regulatory focus (promotion, prevention) - Savings message frame (non-loss, gain) - Brand (well-known, less familiar) - Expiration date restriction (today, two weeks)	- Laboratory - Field	- Hypothetical choice - Choice	Marketing cues can prime regulatory orientations that can reinforce or attenuate a customer’s regulatory focus and influence product choices.
Sales promotions and the choice context as competing influences on consumer decision making. [47]	- Choice set configuration (high-tier national brand and mid-tier national brand and low-tier store brand, high-tier national brand and low-tier store brand, high-tier national brand and mid-tier national brand, mid-tier national brand and low-tier store brand) - Price promotion (for one brand, for none of the brands)	- Laboratory	- Hypothetical choice	Choice set composition drives likelihood of switching between brand tiers due to price promotions.
**In-Store and Customer Decision-Making Factors** **(e.g., Repeated Choices, Customer Goals, Budget Constraints, Payment Methods)**				
**Article Title (Reference)**	**Manipulated Variables (Conditions)**	**Experiment**	**Outcome Variables**	**Key Findings**
The unhealthy = tasty intuition and its effects on taste inferences, enjoyment, and choice of food products. [48]	- Enjoyment goal (primed, not primed)	- Laboratory	- Choice	Unhealthy foods are perceived to be tastier, are rated as more enjoyable during consumption, and are preferred in a choice task when an enjoyment goal (vs. no goal) is primed.
BYOB: How bringing your own shopping bags leads to treating yourself and the environment. [49]	- Bringing reusable bags (yes, no) - Attribution (store, self)	- Laboratory	- Hypothetical choice - Purchase intentions	Customers who bring their own bags to stores are more likely to make unhealthy purchases if they can attribute bringing their own bags to themselves and not the store.
Repeated choosing increases susceptibility to affective product features. [50]	- Choice condition (choice, no choice)	- Laboratory	- Choice	Repeated choice can make consumers more prone to focusing on affective product features.
Smart shopping carts: How real-time feedback influences spending. [51]	- Budget constraint (no budget constraint, budget constraint) - Real-time spending feedback (unavailable, available)	- Laboratory - Field	- Hypothetical choice - Choice	For budget-constrained customers, real time spending feedback leads to higher spending and more overall and hedonic products purchased. For non-budget-constrained customers, real time spending feedback reduces spending but does not have an effect on the number of products purchased.
How credit card payments increase unhealthy food purchases: Visceral regulation of vices. [52]	- Payment mode (credit card, cash) - Product type (vice, virtue)	- Laboratory	- Hypothetical choice	Using credit card (vs. cash) can lead to unhealthier product purchases.
“I Eat Organic for My Benefit and Yours”: Egoistic and altruistic considerations for purchasing organic food and their implications for advertising strategists. [53]	- Focus (egoistic, altruistic, both egoistic and altruistic, control)	- Laboratory	- Purchase intentions	Egoistic, self-focused (e.g., personal health) and altruistic, others-focused (e.g., environmental) considerations influence purchase intentions for organic food.
Seeking Freedom through variety. [54]	- Aisle width (narrow, wide)	- Laboratory	- Choice	Confinement or feelings of confinement can lead to more varied choices.
The power of simplicity: Processing fluency and the effects of olfactory cues on retail sales. [55]	- Scent (simple, complex, none)	- Laboratory	- Hypothetical choice	Simple (vs. complex or no) scents lead to more money spent and more purchases from extra (vs. required) categories.
